# Comparing Clustering Methods Applied to Tinnitus within a Bootstrapped and Diagnostic-Driven Semi-Supervised Framework

**DOI:** 10.3390/brainsci13040572

**Published:** 2023-03-28

**Authors:** Robin Guillard, Adam Hessas, Louis Korczowski, Alain Londero, Marco Congedo, Vincent Loche

**Affiliations:** 1 CNRS, Grenoble INP, GIPSA-Lab, University Grenoble Alpes, 38000 Grenoble, France; 2 Siopi, 15 rue des Halles, 75001 Paris, France; 3 Service ORL et CCF, Hôpital Européen G.-Pompidou, AP-HP, 20, rue Leblanc, 75015 Paris, France; 4 Service d’ORL, Hôpital Claude Huriez, CHU Lille 59000, France

**Keywords:** tinnitus, semi-supervised clustering, subphenotype, bootstrap, benchmark, expert validation

## Abstract

The understanding of tinnitus has always been elusive and is largely prevented by its intrinsic heterogeneity. To address this issue, scientific research has aimed at defining stable and easily identifiable subphenotypes of tinnitus. This would allow better disentangling the multiple underlying pathophysiological mechanisms of tinnitus. In this study, three-dimensionality reduction techniques and two clustering methods were benchmarked on a database of 2772 tinnitus patients in order to obtain a reliable segmentation of subphenotypes. In this database, tinnitus patients’ endotypes (i.e., parts of a population with a condition with distinct underlying mechanisms) are reported when diagnosed by an ENT expert in tinnitus management. This partial labeling of the dataset enabled the design of an original semi-supervised framework. The objective was to perform a benchmark of different clustering methods to get as close as possible to the initial ENT expert endotypes. To do so, two metrics were used: a primary one, the quality of the separation of the endotypes already identified in the database, as well as a secondary one, the stability of the obtained clusterings. The relevance of the results was finally reviewed by two ENT experts in tinnitus management. A 20-cluster clustering was selected as the best-performing, the most-clinically relevant, and the most-stable through bootstrapping. This clustering used a T-SNE method as the dimensionality reduction technique and a k-means algorithm as the clustering method. The characteristics of this clustering are presented in this article.

## 1. Introduction

Tinnitus can be defined as “the conscious awareness of a tonal or composite noise for which there is no identifiable corresponding external acoustic source” [1]. It is a debilitating symptom that affects 14% of the adult population, 2% experiencing a severe form of it [2]. Tinnitus can have disastrous effects on the quality of life of people suffering from it [3]. One of the main characteristics of this symptom is its intrinsic heterogeneity [4], a challenge that is currently being tackled by multiple coordinated efforts especially in the European research community, through the Tinnitus Database initiative [5], the European School on Interdisciplinary Tinnitus Research (ESIT) [6], and recently, the Unification of Treatments and Interventions for Tinnitus Patients (UNITI) project [7].

It has been suggested that tinnitus heterogeneity is partially responsible for the lack of significant treatment outcomes in various clinical trials for tinnitus [5,8,9,10]. Furthermore, several sources advocate for stopping seeing tinnitus as a symptom that would admit a one-size-fits-all solution [11,12,13]. Moreover, it has been suggested that tinnitus interventions might prove to be more specifically effective for particular subtypes of tinnitus [14]. A tinnitus subtype refers here to a part of the tinnitus population with a condition that can be used to guide treatment decisions [15,16].

Likewise, there are convergent opinions on the fact that the observed tinnitus heterogeneity derives from the heterogeneity of underlying mechanisms, potentially cumulative [17,18,19]. In fact, research to identify the underlying pathophysiological mechanisms has led to the identification of several distinct etiologies accounting only for a part of the tinnitus population [20]. Such endotypes (parts of a population with a condition with distinct underlying mechanisms [15,21,22]) are close to having reached consensus. Those endotypes can be diagnosed through specific medical examinations: otosclerosis [23], vestibular schwannoma [24], pulsatile tinnitus [25]. As a consequence, the quest to obtain a tinnitus endotype segmentation appears as a partially solved puzzle [20]. It is interesting to note that, for some cases, the link between phenotypes and the potential associated pathophysiological mechanism is a matter of intense debate. For example, although it seems that Menière’s disease and hydrops (which can now be objectively diagnosed [26]) are linked, the relation between the two entities is not totally bijective and is still being discussed [27].

Various methods have been attempted to identify a global tinnitus segmentation: defining tinnitus subgroups on the response to a tinnitus treatment [28,29], exploring in a data-driven approach the potential inherent structure of tinnitus symptom profiles (i.e., subphenotypes) [30,31,32], highlighting the existence of a particular subtype in a hypothesis-driven fashion [33]. Such attempts often use algorithmic methods designated as clustering to try to infer such segmentations [15,34]. Tinnitus clustering was performed either on the basis of questionnaire information [30,34], medical imaging [35,36], or audiological explorations [31,37], and more rarely, tinnitus clustering has been performed on the basis of free text contents on social networks through a natural language processing method [38,39].

An important methodological limitation to such studies is the limited supervision or the absence of supervision used by those clustering methods to compare their outputs to one another. This makes it difficult to concretely define what makes a clustering closer to an ideal segmentation. At best, some studies have used clusters’ silhouette as the clustering quality criteria [30,40]. In addition, few of those studies have large sample sizes, this being another limitation [41,42].

More fundamentally, this raises the question of the definition of the ideal segmentation. Such clusterings create subphenotype segmentations that would convey limited practical clinical value in and of themselves unless they aimed to obtain as similar as possible either the optimal subtype segmentation or the optimal endotype segmentation. Here, subphenotype refers to a segmentation based on the distinction between sets of observable characteristics [15,22]. In fact, Genitsaridi legitimately presented in her thesis that nothing proves that the three conceptual segmentations (in subphenotypes, subtypes, and endotypes) are identifiable to one another. To illustrate that, it can be plausible that one treatment can work for two different endotypes or that two different endotypes express the same phenotypes or even that, according to individuals, a unique endotype presents itself with varying phenotypes (as for example the subphenotype of tinnitus with otosclerosis is probably not the same before and after surgical intervention).

Table 1, quoted as an excerpt of [43], enables anchoring this important semantic distinction.

In this study, the main objective was to establish a tinnitus subphenotype clustering that would get as close as possible to the ideal tinnitus endotypes’ segmentation. To achieve this goal, the fact that the tinnitus endotype segmentation is already a partially resolved problem, as some etiologies are already known (Menière disease, otosclerosis, etc.), was exploited. This was performed within an original semi-supervised framework to drive the evaluation of clusterings’ quality. Thus, setting the resolution of this problem within this partially labeled framework enabled a quantitative comparison and, thus, a proper benchmark of different algorithmic methods.

## 2. Materials and Methods

### 2.1. Population and Data Acquisition

The investigated database initially comprised 3703 entries of a case history questionnaire recorded by tinnitus patients over the last 20 years of practice by V.L., ENT specialist at the Lille University Hospital, France. Entries were paper-filled then computer-recorded and carefully anonymized before being compiled for analysis in a comma separated value (csv) format file. Patients gave their informed consent that their collective entries could be aggregated, anonymized, and then, used for clinical research purposes.

Features of the database included demographic variables (age, sex, etc.), characteristics of the tinnitus (side, frequency, loudness, etc.), subjective visual analog scales measuring the impact of tinnitus on patients’ quality of life (on anxiety, sleep disturbance, etc.), information on patients’ lifestyle (sleep duration, practice of sports, etc.), as well as, whenever possible, the diagnosis of V.L. Table 2 and Table 3 summarize the main features characteristics over the preprocessed database.

This database offers the advantage of gathering a variety of tinnitus-related descriptors, as well as, when possible, the medical diagnosis given by an expert medical doctor. The latter offers the opportunity to test diagnostic-driven semi-supervised approaches of clustering.

Only a minority subset of the dataset was labeled with a diagnosis by the expert doctor. Some endotypes were less represented than others, reflecting their unequal prevalence in clinical practice. The different endotypes considered in this study and their prevalence in the dataset are presented in Table 4.

### 2.2. Preprocessing

Several preprocessing steps were needed to make the raw data exploitable. Such steps are described hereafter. Pre-processing steps were conducted with the objective to limit the introduction of additional bias in the dataset.

#### 2.2.1. Missing Value Imputation

Case history questionnaire collection was paper-based before being computer-recorded. Such a procedure induced, at both steps, missing values in the raw dataset. Missing values’ imputation is a classic problem in data analysis and machine learning [44,45]. Mainly, two methods have been proposed to remediate this issue: removing or replacing the missing values. The main objective of missing value management is to limit as much as possible the introduction of additional bias in the dataset while keeping a maximum of patients and features available for analysis. To meet these two objectives, the following method was implemented:

- First, each patient that had more than 40 percent of missing values in the initial 107 features was deleted to ensure overall data consistency. Through this procedure, out of the 3703 initial patients, 2772 were retained (74.9% of the initial 3703 patients sample).
- Then, for each feature, if the number of patients for which the feature was missing was higher than 25 percent, the feature was excluded from the analysis. Through this procedure, out of the 107 features considered, 44 were retained (41% features retained).
- Filling the remaining missing values using the MissForest algorithm, specially designed to fill missing values with an iterative imputation method based on a random forest [46].

At the end of this process, the final dataset no longer contained any missing values and consisted of 2772 patients and 44 features.

#### 2.2.2. Bootstrap Procedure

With the aim to perform an objective comparison of performances between combinations of dimensionality reduction methods and clustering algorithms, stability assessment of the clusterings was performed. This stability assessment was realized through a bootstrap procedure. The dataset was split into a training set and a test set three times, with a resampling with replacement procedure. Such partitionings all contain the entirety of the dataset, split randomly in different ways according the procedure described hereafter.

In classical cross-validation practice, the design of such partitionings results in a higher percentage of data attributed to the training set and a lower proportion to the test set. In the case of the present study, the t-distributed stochastic neighbor embedding (t-SNE) method was included in the benchmark. This method relies on the high-dimensional topological structure and local density of the data. Due to this fact, having significantly different numbers of samples in the training sets and test sets would result in an increased probability of divergence in the high-dimensional spatial distribution of samples and, as a consequence, an increased divergence in the projection spaces. It was, hence, necessary for this method to have a 50-50 split of the dataset. Hence, we could not perform a classic cross-validation procedure to be able to compare all combinations of methods tested in the benchmark in the same conditions. This is why it was decided instead to bootstrap three 50-50 partitionings between the training and test sets for all combinations of methods.

As the evaluation of clustering performances is based on the diagnoses of an ENT tinnitus expert, it was important that each endotype was equally represented in each partitioning of the data between the training and test subdatasets. Hence, a random assignment procedure was applied to each endotype and the unlabeled data group to equally divide them between the three bootstrapped training and test subdatasets. Following the presented results are the average of the performances between the three partitionings of the dataset.

### 2.3. Dimensionality Reduction Methods

Even after excluding features with missing values, the remaining number of variables is important and justifies the use of dimensionality reduction methods to achieve the best performances of the clustering algorithms.

There are methods that try to reduce the number of features ahead of the clustering method while trying to preserve as much information as possible from the initial dataset. In this study, three different dimensionality reduction methods were used and compared: PCA [47], factor analysis of mixed data (FAMD) [48], and finally, t-distributed stochastic neighbor embedding (t-SNE) [49].

The number of dimensions was tested repeatedly for a defined range of values and treated as a hyperparameter, thus validating a posteriori the quality of the dimensionality reduction method.

Each of the three methods is hereafter described in further detail.

#### 2.3.1. Principal Components Analysis

PCA is a classical dimensionality reduction technique that is typically used to summarize large datasets with a small number of descriptors while retaining the maximum amount of information. PCA does not aim at selecting the best characteristics while dropping others; instead, it constructs some new characteristics named principal components, based on the original features of the dataset. More formally, PCA is a linear dimensionality reduction technique that creates a set of pairwise orthogonal axes that maximize the variance in the data. Thus, this method helps remove redundancy in the new reduced feature space.

#### 2.3.2. Factor Analysis of Mixed Data

PCA was designed for quantitative and non-categorical data analysis. However, our dataset is composed of a mix of categorical and non-categorical variables. FAMD was chosen for our benchmark of dimensionality techniques as it is similar to PCA, but takes into account the mixed aspect of the data. The implementation used is reachable in the Python library Prince (https://github.com/MaxHalford/prince, accessed on 23 November 2022).

#### 2.3.3. t-Distributed Stochastic Neighbor Embedding

The last two methods allow an analysis of the global structure of the data, but do not provide an insight into the local topological structure of the data points. Given the objective to create an accurate clustering of endotypes for tinnitus, the local grouping of data points may be of particular importance. Thus, a third dimensionality reduction technique was considered.

The dimensionality reduction algorithm called t-distributed stochastic neighbor embedding (t-SNE) is an unsupervised learning algorithm. Developed by Laurens van der Maaten and Geoffrey Hinton [50], it enables the analysis of data described in high-dimensional spaces (via a large number of descriptors) to be mapped into a reduced dimensional space. This algorithm is widely used because it facilitates the visualization of data with many descriptors. Through this method, data points that are close in the original high-dimensional space have a higher probability of remaining close to one another in the two- or three-dimensional space of projection. Conversely, data points that are distant in the original space have a low probability of having close representations in the space of projection.

There is a hyperparameter that has a strong impact on the output of the t-SNE algorithm: the perplexity. It characterizes the balance of importance between the local neighborhood structure versus the global neighborhood structure of the data. A large perplexity will lead the algorithm to put the emphasis on the global neighborhood structure of the data. Oppositely, a small perplexity will bring out the local structures of the original data. In this study, the t-SNE method was tested for a wide range of perplexity values (from 5 to 200).

### 2.4. Clustering the Dataset

In this study, a clustering step was applied after the dimensionality reduction. Thus, the best clustering will be selected, and the adequate parameters to reduce the dimensionality will be inferred from it.

Two different clustering algorithms were compared: the k-means clustering algorithm and density-based spatial clustering of applications with noise (DBSCAN) clustering algorithm [51].

#### 2.4.1. k-Means Clustering

k-means is a clustering algorithm that aims at partitioning a dataset of observations into k clusters: each data point is attributed to the cluster with the nearest mean. It is a classic method of clustering that has already been tried in several former studies for tinnitus subphenotyping [34,52]. The three main assets of this method are its easiness of interpretation, simplicity of implementation, and speed of convergence. Due to the nature of this study, the number of clusters was not predefined. Yet, it is necessary to specify to the algorithm the number k of clusters to find for it to be able to run; a range from 2 to 20 was hence implemented for the grid search (as shown in Table 5). The distance measure on numeric attributes was the square Euclidean distance.

#### 2.4.2. Density-Based Spatial Clustering of Applications with Noise

DBSCAN is a non-parametric clustering algorithm that takes a given set of points in an initial space, then groups together points that are densely packed together (points with many nearby neighbors), and marks as outliers points that are isolated in low-density regions (whose nearest neighbors are too far away).

It was selected for its different characteristics from k-means: it is a density method and is able to find arbitrarily shaped clusters, while k-means produces Voronoi-cell-shaped clusters. DBSCAN has been used once to try to perform tinnitus subphenotyping [53].

DBSCAN exploration is driven by two hyperparameters: The first is minsamples, the minimal number of samples (or total weight) in a neighborhood for a point to be considered as a core point. This includes the point itself. Second is eps (standing for epsilon): the maximum distance between two samples for one to be considered as being in the neighborhood of the other. The ranges of exploration of these hyperparameters for the grid search are shown in Table 5.

### 2.5. Quantified Evaluation of the Quality of the Clusterings

The following procedure was applied for the benchmark. For each type of clustering and dimensionality reduction technique tested, the best hyperparameters for clustering were identified through grid search exploration, presented in Table 5. Six combinations of dimensionality reduction techniques and clustering methods were, hence, tested and compared according to the different mathematical methods presented hereafter.

Then, a shortlist of the best clusterings was obtained according to these mathematical criteria and was then presented and analyzed by two tinnitus ENT experts to check their clinical relevance. Out of this comparison, a single clustering was selected and is presented hereafter.

To check the quality of the clusterings, three separate quantified evaluation criteria were used.

#### 2.5.1. Silhouette Score

The “silhouette measure of cohesion and separation” is a measure for the overall goodness-of-fit of the cluster structure, which is described in [54,55]. More precisely, the silhouette value is a measure of how similar an object is to its own cluster (cohesion) compared to other clusters (separation). The silhouette ranges from −1 to +1, with a high value indicating that the object is well matched to its own cluster and poorly matched to neighboring clusters. If most objects have a high value, then the clustering configuration is considered appropriate. If many points have a low or negative value, then the clustering configuration may have too many or too few clusters. Due to its construction, this score will advantage the clustering algorithm that will tend to form spherical-like clusters, such as k-means, over less regularly shaped clustering methods, such as DBSCAN. Moreover, its interpretation is less obvious in the framework of the dimensionality reduction t-SNE algorithm used in this study. Hence, this score will only be shown because it is a classical way to evaluate clustering and was used in a former study on tinnitus clustering [30], but will not be eventually determinant for the choice of the final clustering.

#### 2.5.2. Stability Assessment

For each combination of the dimensionality reduction techniques and clustering algorithms, it is necessary to ensure the stability of the clusterings obtained. To achieve that, a bootstrap procedure was designed.

It is common for artificial structures to emerge that do not correspond to the real separation of the data. In the case of the present study, an output would be deemed stable through bootstrapping if the structure of the clusterings obtained through the application of the same method with the same parameters on the training and test sets of the data are similar. The similarity between two clusterings, which can be identified for two data partitions, can be reliably measured by the adjusted mutual information (AMI) [56]. This measure was, hence, selected for this study.

To measure the agreement of two data partitions (i.e., clusterings) *U* and *V*, the AMI takes a value of 1 when the two partitions are identical and 0 when the AMI between two partitions equals the value expected due to chance alone. Its calculation is implemented by the following formula:

(1) $$AMI\left(U,V\right)=\frac{MI(U,V)-E\{MI(U,V)\}}{\max\left\{H(U),H(V)\right\}-E\{MI(U,V)\}}$$

The bootstrap procedure was the following: Given a partitioning i of the data (between 1 and 3), the clustering method is fit on the (already projected in reduced dimension) training and the test sets. The prediction method of the two obtained models is then applied to the training and the test set. The output of this procedure is four clusterings: two clusterings on the training set (the one fit on the training set and the one fit on the test set) and two clusterings on the test set (the one fit on the training set and the one fit on the test set).

The AMI was applied between the two pairs of clusterings stemming from the same data (i.e., respectively between the two clusterings of the training set and between the two clusterings of the test set). The average of two scores’ AMI was then taken as the final score.

This method was applied for the k-means clustering, yet it was not possible to apply it for the DBSCAN method, as it is a transductive method; hence, in this procedure, the fitting method cannot be disentangled from the prediction method. In this case, a proxy of cross-validation was performed by calculating the difference of the number of clusters between the training and test clusterings’ outputs of the DBSCAN applied with the same hyperparameters.

Here, an eligibility threshold of at least 0.7 for the averaged AMI score and a difference of 0 between the number of clusters emerging from the training and test sets were applied to filter out the best solutions of the benchmark.

#### 2.5.3. Clustering Similarity to the Endotype Labeling Clustering Enabled by the Partial Medical Diagnosis of the Patients

As a key goal of this study was to take advantage of the fact that the endotype segmentation is a partially resolved problem, the quality of the obtained clusterings was evaluated through this partial knowledge. The dataset considered contains, whenever possible, a diagnosis of the endotype of the patient provided by an ENT specialist (V.L.).

The optimal clustering of the whole dataset would be a clustering that would be at least able to separate each of the known endotypes and assign it to a separate cluster. In order to be able to quantify how close a clustering is to an optimal clustering of reference, a metric is needed. Such an evaluation criterion is given by the V-measure, an entropy-based cluster evaluation measure, presented by Rosenberg and Hirschberg [57].

The V-measure between a clustering considered as the reference and a clustering obtained as the output of an experimental algorithmic procedure is defined as the weighted harmonic mean of two other metrics called homogeneity (*h*) and completeness (*c*) and is given by the following formula:

(2) $$V-measure=\left(1+\beta \right)*\frac{h*c}{\beta *h+c}$$

where β is a hyperparameter ∈[0,∞], quantifying the relative importance of homogeneity and completeness. The V-measure score is bounded between 0 and 1, where a score of 1 corresponds to a perfectly complete and homogeneous matching between clusterings. If β≤1, the emphasis is on homogeneity. Otherwise, completeness is highlighted. One can note the analogy between this metric and the F-score used in classification and being composed of the precision and recall. A clustering result is homogeneous when all of the clusters it formed contain only data points that are members of a single class. A clustering result satisfies completeness if all the data points that are members of a given class are elements of the same cluster.

In this study, we wanted to isolate to the best of our ability the known endotype clusters. After careful examination of the results produced by various β values, β=0.1 was chosen as the better proxy.

**Figure 1:**
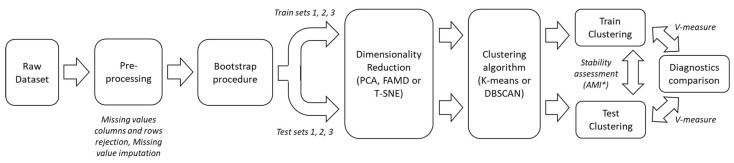
Overview of the processing pipeline.

The whole process is summarized in Figure 1. The range of exploration for hyperparameters used for the grid search is presented in Table 5.

### 2.6. Qualitative Evaluation of the Obtained Clusterings

The grid search exploration of all hyperparameters for all combinations of the method of the benchmark and their ranking using the evaluation criteria led to a subset of best-performing clusterings. To be able to present the overall best-achieved clustering obtained by this study, a last step of qualitative evaluation was achieved by ENT tinnitus experts A.L. and V.L.

The subset of the three overall best-performing clusterings obtained at the end of the quantitative evaluation procedure was selected and presented for the quantitative evaluation of the appointed experts.

**Figure 2:**
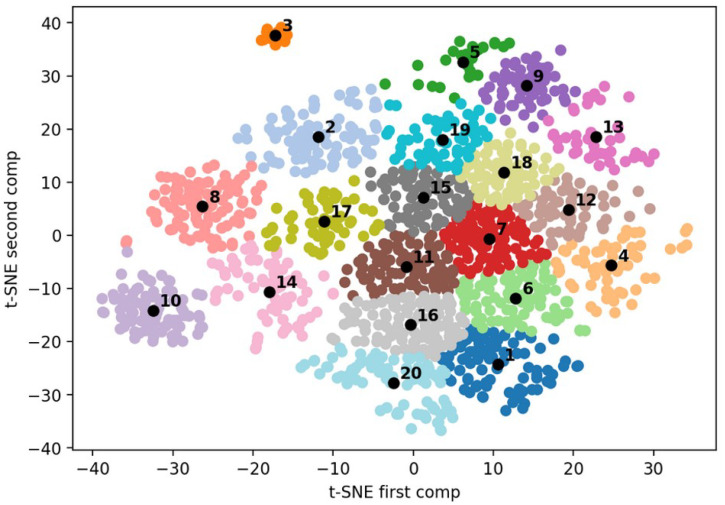
Selected clustering representation of the two-dimensional t-SNE projection. The black points represent each of the clusters’ centers (i.e., their centroids). The numbers are the labels of each cluster. Table 6 refers to these cluster numbers to characterize each of the clusters.

Each clustering was presented with the following information: the combination of hyperparameters and methods leading to this clustering, an illustration of the clustering in the reduced dimensionality projection of the dataset when possible (as in Figure 2 of the article), a table presenting the specific characteristics of each clusters.

The procedure of the qualitative evaluation of the clusterings was performed by each expert independently. The evaluation consisted of assigning a score between 0 and 5 for each cluster of each clustering. A score of 0 means that the cluster appears incoherent and does not relate to anything encountered in their clinical practice, and a of score 5 means that the cluster fits perfectly a well-known and potentially documented specific subphenotype of patients often encountered in clinical practice. Then, a general appreciation of each clustering was freely given to each clustering.

The final score for each clustering was obtained by averaging the scores for all clusters and for the two experts.

**Figure 3:**
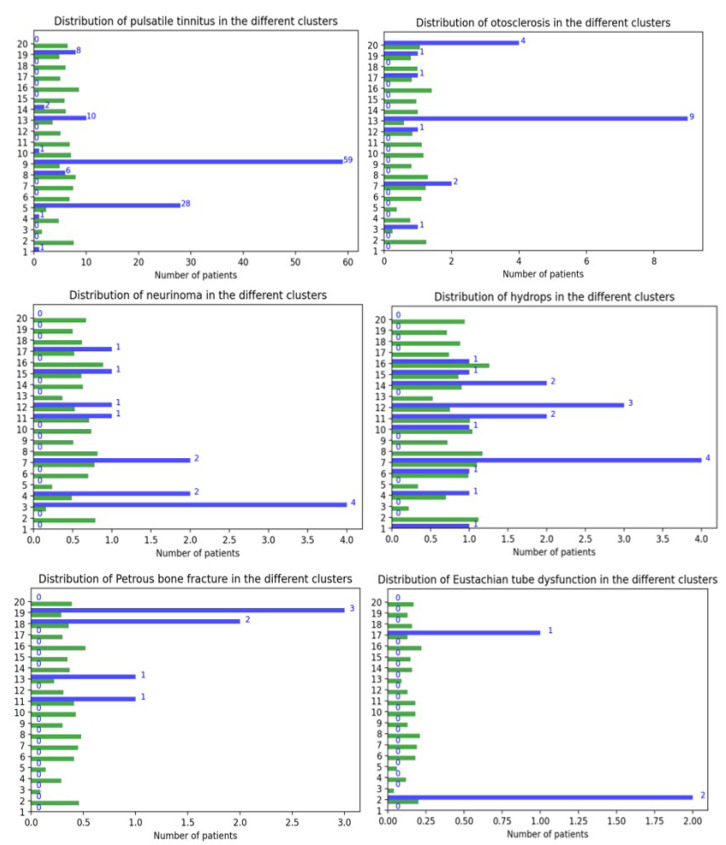
Endotypes’ repartition. The blue bars represent the number of samples of a specific endotype present in the cluster of the number it faces. The green bars represent the number of samples of the same specific endotype that this cluster would contain if the patients of this endotype were randomly assigned to the clusters. For example, on the top left, Cluster 9 contains 59 patients presenting a pulsatile tinnitus, although if the assignment of pulsatile tinnitus patients per cluster was random, we should have expected to have between five and six pulsatile patients in this cluster.

The final best clustering obtained by this procedure is presented in the Results Section, by Figure 2 and Figure 3 and Table 7.

## 3. Results

### Results Overview

Table 6 presents the results of the best-achieved performances for each combination of methods of the benchmark explored in this study, for the evaluation metrics presented in the methods.

The best performances on all three evaluation criteria were achieved simultaneously by the combination of the t-SNE dimensionality reduction technique and the k-means clustering algorithm. These performances were evaluated on the averaged performances on the three partitionings of the dataset obtained by the stratified random subsampling procedure. A set of three best-performing clusterings was constituted at the end of this procedure and was then presented for the quantitative evaluation of the two ENT tinnitus experts.

The three best clusterings were obtained from the combination of the t-SNE dimensionality reduction technique and the k-means clustering algorithm. The sets of hyperparameters of these three clusterings were: Clustering 1: perplexity = 40, number of components for t-SNE: 2, number of clusters of the k-means: 20; Clustering 2: perplexity = 75, number of components for t-SNE: 2, number of clusters of the k-means: 20; Clustering 3: perplexity = 5, number of components for t-SNE: 2, number of clusters of the k-means: 18. The three clusterings presented to the ENT tinnitus experts were the ones of the partitionings having the best V-measure score for the given set of hyperparameters.

The overall averaged qualitative scores between the ENT Tinnitus experts’ evaluations gave the following scores: Clustering 1: 4.18/5, Clustering 2: 4.53/5, Clustering 3: 4.22/5.

The best-performing clustering at the end of this procedure was Clustering 2 with hyperparameters” perplexity = 75, number of components for t-SNE: 2, number of clusters of the k-means: 20. Both ENT tinnitus experts ranked this clustering as the most-clinically relevant.

As this clustering is projected in a two-dimensional space by the t-SNE method, it was possible to present it in Figure 2. The details of the characteristics of each cluster of this clustering are presented in Table 7. The endotypes’ repartition associated with this clustering is presented in Figure 3.

## 4. Discussion

In this study, the main objective was to establish a tinnitus subphenotype clustering (i.e., a clustering of parts of a population with a distinct set of observable characteristics [22,43] that would get as close as possible to the ideal tinnitus endotype segmentation (i.e., a segmentation between parts of a population with a condition with distinct underlying mechanisms [21,43]. To achieve this goal, a bootstrapped semi-supervised and diagnostic-driven benchmark of combinations of algorithmic methods was performed to obtain the best-possible clusterings of a given dataset of tinnitus patients. The final choice presented in the results was selected qualitatively by ENT tinnitus experts among almost equally performing clusterings on the basis of its most-accurate clinical relevance. The best clusterings were performed by a combination of t-SNE dimensionality reduction and k-means clustering and successfully separated the known endotypes of tinnitus within different clusters. The important number of clusters (18 to 20) of the final subset of best-performing clusterings highlights the clinically observed and reported highly heterogeneous nature of tinnitus [4]. The obtained subphenotypes yielded interesting bases for further explorations of the underlying pathophysiological mechanisms of tinnitus on specific tinnitus homogeneous subpopulations of patients. This could facilitate the discovery of new endotypes of tinnitus.

### 4.1. Final Clustering Description

As the finally selected clustering was projected in a two-dimensional space by the t-SNE dimensionality reduction step, it can easily be displayed and analyzed. It is important to specify that the presented clustering was applied on the training set of the third partitioning of the data. This is the reason why only half of the whole sample of patients is included in it.

The general spatial organization of this clustering, as well as the two others that were evaluated is composed of a big core (here, the grouping of Clusters 1, 4, 6, 7, 11, 12, 15, 16, 18, 19, and 20) and gravitating satellites (Satellite Clusters 2, 3, 5, 8, 9, 10, 13, 14, and 17).

Only the finally selected clustering is presented in this study, yet it is important to note that some regularities were observed in all three clusterings presented for the evaluation by the ENT Tinnitus experts:

- The cophosis cluster (Cluster 3) was always dense and isolated in all clusterings. These patients have most probably a unilateral cophosis, and it makes sense that it contained neurinoma patients (probably post neuro-radiological procedure).
- The high frequencies of hearing loss and tinnitus cluster (Cluster 10), the pulsatile group composed of Clusters 5 and 9, and the somatic group composed of Clusters 8 and 14 were always satellites of the clusterings. The pulsatile and somatosensory groups were sometimes partitioned in two as in this clustering and sometimes unified within only one cluster.
- The main core group of patients had always a gradient structure where, at one extremity, the impact of tinnitus and associated symptoms is very important at one end (as in Clusters 12, 15, 18, and 19) and the impact is either mild or absent at the other end (patients not impaired as in Clusters 1, 16, and 20). These “poles” have an influence on the general spatial organization: the otosclerosis Satellite Cluster 13 where the patients are symptomatic and annoyed was close to Clusters 12 and 18, and similarly, non-disturbed somatic tinnitus patients Cluster 14 was close to Clusters 16 and 20. It is worth mentioning that the gradient of impact on the quality of life of tinnitus was almost aligned and coincident with the gradient of associated quality of sleep measured by ISI, VAS sleep quality, sleep latency, nocturnal awakenings, etc. In fact, sleep was disturbed in Clusters 7, 12, 15, 18, and 19 in opposition to Clusters 1, 14, 16, and 20 (except that, in Cluster 20, patients have OSA, but do not seem to be disturbed by it).
- This clustering seemed to highlight and isolate clusters centered on sudden hearing loss (Cluster 11) and head trauma (Cluster 19) and to show an association between hyperacusis and headaches (Clusters 15 and 17). It was also interesting to observe that one of the pulsatile tinnitus clusters had the feature “side right” presenting an important effect size. In fact, it is reported in the literature that, very often, the tinnitus is localized on the right side, due to the important prevalence of venous origin pulsatile tinnitus in the population [58]. Likewise, it is no surprise that, for both pulsatile tinnitus groups, effect sizes highlighted a over-prevalence of women in these groups, as well as lateralized tinnitus.

### 4.2. Merits

#### 4.2.1. Changing Framework from Non-Supervised to Semi-Supervised Enabling Benchmark on Performance

This study was the first in the field of tinnitus to leverage the partial existing knowledge of tinnitus heterogeneity to drive the clustering procedure. Although this procedure can probably be largely improved in the future (see the Limits Section and the Suggestions for Future Research Section), it opens the path to a new framework of analysis on the issue of tackling tinnitus heterogeneity.

The main perspective that this new framework brings for future research is a (partial) ground base for evaluating the performance of tinnitus patient segmentation (and in the case of the present study, tinnitus clustering). This basis enables quantitatively evaluating and, thus, comparing the performances between different competing algorithmic methods or a combination of methods to solve the task at hand. For the first time, a benchmark of performances between several combinations of methods was made possible to evaluate which combination best reproduces the partially known diagnostic segmentation. It should be noted that the best-performing clustering obtained in the present study achieved a 0.386 score on the main criterion, the V-measure score. Such a score is comparable to the best performances achieved for spondyloarthritis clustering by a recent study with a similar database size of 3438 patients. The best clustering of this study obtained a V-measure score of 0.588 [59].

#### 4.2.2. Stability Assessment

This study is also the first to have performed an equivalent to a cross-validation procedure (here, a bootstrap procedure) aiming at ensuring the quantified stability of its clusterings, following the suggestion of [15]. Here, three partitionings of the dataset into training and test subdatasets were conducted following a resampling with replacement procedure. The evaluation of stability involved the use of the adjusted mutual information score between clusterings, as described in the stability assessment section.

Applying such a procedure is a well-known safeguard against over-fitting of the model and ensures its replicability to some extent.

It is also important to mention that, in the present study, adjusted mutual information scores between the best-performing clusterings were surprisingly quite elevated (>0.7), suggesting a quite stable structure of the dataset.

#### 4.2.3. Performing Clustering on a Large and Rich Patient Questionnaire Sample

Such semi-supervised paradigm adoption would hardly have been possible without the importance of the unified dataset analyzed in this study. It is also important to stress that the 44 features reported per sample in this database covered a wide spectrum of tinnitus patient characteristics from hearing, hyperacusis, to somato-sensory modulations and to associated sleep disturbance and depression. Such a specter covers the majority of items suggested to be addressed by the review on tinnitus clustering published by [15].

#### 4.2.4. Reproducing in the Benchmark Already Tested Dimensionality Reduction Methods and Clustering Methods and Proposing Original Combinations

In the present study, the methods (PCA, k-means, and DBSCAN) used by [52,53] were included in the benchmark and applied on the same dataset. Likewise, the silhouette score evaluation method presented in [30] was reproduced and presented, although it was not the main criterion for discriminating the performances of the clusterings.

New original methods have also been proposed and included in the benchmark of this study: One was the factor analysis for mixed data framework that appeared as a promising improvement to the classic PCA approach. It actually performed slightly better than PCA when combined with either k-means or DBSCAN. Likewise, t-SNE appeared as a relevant dimensionality reduction technique to be applied on such mixed data and performed best in combination with the k-means clustering method.

### 4.3. Limits

#### 4.3.1. Missing Values

A first limit of this study was the non-negligible presence of missing data in the initial dataset, which required excluding some features, as well as a non-negligible amount of patients (929 rows) and the use of missing value imputation. Although an adaptive and stable method was used for missing value imputation (MissForest [46]), even more accurate results could have been obtained with a complete and larger dataset.

#### 4.3.2. Limited Number of Diagnostics

The main aim of the study was to create a clustering that would find and separate the known endotypes of tinnitus. Yet, due to the retrospective nature of this work performed on an already acquired dataset, several limitations are to be considered on this matter in the present study. First, although this labeling was performed by a skilled ENT specialist and on the basis of objective diagnostic measurement, it cannot be excluded that some diagnoses were wrongly attributed to some patients. Even more, some patients were probably not diagnosed, although they were part of a known endotype. The diagnosis partitioning that served as a reference in this study is hence limited in its validity.

Secondly, the prevalence of some endotypes is naturally rare in the tinnitus population. This dataset was obtained over several years of clinical practice taking care of any patient that sought the help of this practitioner. Consequently, there has been no specific selection procedure applied that would have led to a more advantageous representation of known endotypes in the dataset. As a consequence, the total percentage of labeled data in the dataset was quite low (12.59%), and the prevalence of some endotypes was extremely low (seven patients for Eustachian tube dysfunction).

Lastly, a question that remains open is the boundary between what can be considered as a “known endotype” and a “comorbidity”. It could be argued that “ototoxicity”, a good potential candidate, should not be included in such work as several different drugs could lead to such an attribution of the label without affecting similarly the auditory system. Yet, the question is posed when it comes to more prevalent subgroups such as presbyacusis, acoustic trauma, somato-sensory tinnitus, or sudden hearing loss to a lesser extent: Should such conditions be considered as endotypes of tinnitus? More refined criteria to delimit between what should be considered eligible labels or comorbidity should be proposed in the future.

On the other hand, some etiologies chosen in the present study could be considered as heterogeneous. For example, pulsatile tinnitus patients all received the same diagnostic label, although it could be argued that one should have separated patients affected by semicircular canal dehiscence, carotid aneurysm, neurovascular conflict, etc. This example highlights the question of the level of granularity one should give to the definition of a tinnitus etiology, a question that goes beyond the scope of the present work. Here, the decision to take pulsatile tinnitus as a unique diagnostic label was a pragmatic choice acknowledging the limited number of labels available to map such pulsatile tinnitus sub-etiologies. The limited size of the database in and of itself also conditioned the maximum number of clusters that could be allowed for the grid search. Moreover, only a weak constraint from the V-measure was placed on the algorithms on the number of clusters that could include pulsatile tinnitus. As a consequence, the best clustering presented in the results exhibited two different clusters with pulsatile tinnitus patients.

#### 4.3.3. Mono-Label Clustering

In this study, a methodological choice was to attribute each patient to one endotype and one endotype only. It was hence a mono-label clustering. Yet, it could be easily argued that this methodological choice over-simplifies the sometimes complex and intricate nature of clinical presentations of some patients. For example, as the ENT tinnitus experts commented on the results of the final clustering, it was reassuring to observe that some “pulsatile tinnitus” patients were attributed to the cluster where otosclerosis was predominant (Cluster 13). Indeed, in some cases, otosclerosis can produce a tinnitus presenting with a pulsatile sound.

#### 4.3.4. Biases of the Questionnaire

There is no ideal questionnaire to make an optimal anamnesis of tinnitus. However, some good directions were given regarding the important components such questionnaire should at least contain so that the chances to perform a good clustering are maximized [15]. Here, due to the retrospective nature of this study concerning the database acquisition, the questionnaire used, although reasonably complete, did not include all the items suggested by this review.

Another more subtle bias in the questionnaire is the heterogeneity in the level of scrutiny given to some dimensions of the symptomatology compared to others. In the case of the questionnaire used for this study, only one feature in the dataset was associated with vertigo (VAS scale on vertigo), whereas eight features were devoted to the impact of tinnitus on sleep (ISI, Epworth, VAS scale on sleep disturbance, nocturnal awakenings, sleep latency, quality of the sleep onset, snoring, sleep apnea). This bias was most probably introduced by the fact that V.L. has studied sleep medicine in addition to his ENT specialization. Such over-representation of sleep features in the dataset, compared to others, induced biases in the dataset that may not have been solved by the redundancy limitations induced by the dimensionality reduction techniques.

It could also of course be argued that the focus given on tinnitus interactions with sleep in the present study brings additional value to this study. Indeed, until now, the description of the nature of those interactions had been limited [60].

#### 4.3.5. Limited Range of the Hyperparameters for the Grid Search

In the present study, the grid search parameters were set to explore a range of values that may have constrained the exploration to a space yielding suboptimal performances. A wider range for grid search was explored in testing the dataset before the decision was made on these ranges. From these explorations, it seems that widening the ranges for the dimensionality reduction hyperparameters’ PCA, FAMD, or t-SNE components, as well as for perplexity for t-SNE did not seem to produce better performances. It was also remarkable to note that the best performances on the V-measure were always obtained on the lowest possible numbers of components (mostly 2, sometimes 3).

Notably, it is unsure whether the ranges explored for minimum sample and epsilon hyperparameters for DBSCAN were optimal. Above all, the range of exploration for the k-means number of clusters was selected as a trade-off and not as the optimal for performance. In fact, simulations of the V-measure’s best performances showed it to be an increasing function of the maximum number of clusters. This function, for the different combinations of algorithms, had an asymptotic convergence. The asymptotic value, as well as the speed of convergence depended on the combination of algorithms, as well as the value of the parameter β of the V-measure. For the different simulations performed, it was observed that the combination t-SNE + k-means, which elicited the best results for this study, was also the combination of algorithms that converged the most rapidly to the asymptotic value and had the most-elevated asymptotic value.

The maximal value of 20 for the range of exploration of the number of clusters was the result of a trade-off: this number was enough to reach 75% of the asymptotic value for this method, while breaking the samples into groups of around 70 samples on average. It made it possible to characterize the symptomatic specificities of each cluster. Likewise, it brought a fine granularity for the evaluation of clusterings for the ENT tinnitus experts and separated the endotypes without dividing each endotype too many times into different groups. What was surprising was that, while simulating the clusterings for a greater number of clusters (for example taking a maximal number of clusters yielding at least 95% of the asymptotic value of the V-measure), the obtained clustering appeared to divide the endotypes too much. This suggests that the V-measure might not be totally optimal in achieving the desired goal. It is important to point out that increasing the value of β instead (to favor less the optimization on homogeneity) resulted in another problem: the results regrouped different (and clinically incompatible) endotypes in the same clusters.

#### 4.3.6. Limited Stability Assessment for the DBSCAN Output

In the present study, a rigorous bootstrap procedure was applied to assess the stability of the k-means clustering method’s output. Yet, such a method could not be applied for the DBSCAN algorithm. In fact, due to the transductive nature of DBSCAN (i.e., it cannot predict the labels of new data), the fit and predict methods of DBSCAN cannot be disentangled, so it was not possible to make a prediction of the labels of a subset of samples with a model fit on the other sample subset. Counting the difference of the clusters in the outputs proposed by the DBSCAN was used as a replacement for the stability assessment: indeed, the number of clusters of the output clustering is a free parameter of DBSCAN. Yet, it can be questioned whether such replacement constitutes the best-possible stability assessment for a transductive method.

### 4.4. Suggestions for Future Research

#### 4.4.1. Widen Hyperparameter Search on Larger Databases

As stated in the Limits Section, our grid search ranges were limited, especially for the maximal number of clusters, which was chosen as a trade-off considering the sample size of the database.

On the other hand, it seems intuitive to induce that if six different endotypes of tinnitus are already present in 12.59% of the dataset as in the present study, it could be expected that the total number of clusters of an ideal clustering should be above 20. Future research should hence work to apply such techniques on wider datasets to enable widening the range of hyperparameters.

Similarly, the set of features of our database was limited and did not cover all the important dimensions advised in [15]. Future research should constitute their databases prospectively and, hence, actively shape the questionnaires so as to cover globally the anamnesis of a tinnitus patient. Focus should also be put on not over-weighting the exploration of some dimensions compared to others (as illustrated in the limits for vertigo and sleep in our case).

#### 4.4.2. Change Framework to Longitudinal Data

A supplementary important suggestion for future research would be to switch from an initial dataset composed of a unique point in time (and thus, a unique completion of a given questionnaire) per patient to a longitudinal dataset in which each patient should answer a given set of questionnaires at some strategic points in time. In fact, the clustering chosen to be presented in this study tried to capture the time dimension by the feature “tinnitus duration”. Yet, such a metric poorly captures the level of intrusiveness of a patient, which can evolve at different speeds according to the psychological and behavioral adaptation to the tinnitus and to the efficiency of therapeutic interventions. Likewise, some clusters tend to reflect such a difference of temporality, with clusters that evoke a state of initial crisis and others where one could hypothesize that patients are habituated to their condition (i.e., where the tinnitus annoyance is significantly lower).

It would naturally require more resources to lead such a study over a longer time frame to enable mapping the trajectories of patient symptoms. However, such initiatives could be led by digital mobile platforms such as TrackYourTinnitus [61] or Siopi [62]. Furthermore, it would require reorganizing the framework of analysis of such a characterization of each patient so as to achieve a clustering of different trajectories of patients, rather than a clustering of questionnaire entries.

#### 4.4.3. Going Further in the Semi-Supervised Framework

In the present study, the semi-supervision of the algorithm only intervened in the evaluation of the clusterings obtained at the end of a systematic unsupervised process set in motion on a constrained grid search exploration space. This is the reason why the description of the procedure is only referred to as a semi-supervised *framework*. Yet, future research should investigate how to include semi-supervision directly into the pipeline of clustering. It could be at the level of the dimensionality reduction or at the level of the clustering algorithms [63,64].

#### 4.4.4. Going from Mono-Label to Multi-Label Clustering and beyond

As suggested among the limits of the present study, the methodological choice of applying a mono-label clustering might not be well fit to describe the clinical reality of patient symptomatology. When defining endotypes more restrictively, it becomes possible for a patient to be part of several endotypes: for example, a patient presenting presbyacusis with somato-sensory tinnitus due to a jaw instability would hence be assigned to two endotypes, as these two reasons in and of themselves can lead to the emergence of tinnitus.

It would hence appear useful for future research to adapt the clustering framework to perform a multi-label clustering.

To go even further, it can be questioned whether segmentation, due to its discrete and non-continuous nature, is the best methodological tool to address tinnitus heterogeneity. Another possibility to address the heterogeneity problem would be to tackle it with a more continuous and local approach by only looking locally for the best neighbors to a given patient. Such an approach has been attempted by Siopi as its mutual-help community could involve metric learning methods (supervised or weakly supervised) such as metric learning with application for clustering with side information (MMC) [65], large margin nearest neighbor metric learning (LMNN) [66], or other deep metric learning methods.

#### 4.4.5. Apply the Semi-Supervised Clustering to Other Labels

It seems important to point out that a semi-supervised framework for tinnitus clustering can also be applied taking as a reference something different from endotype labels. For example, taking individual treatment responses as the labels could also be very interesting to try to define subtypes of tinnitus.

#### 4.4.6. Explore Other Dimensionality Reduction Techniques and Clustering Techniques

Here, methods that had been tested in past studies were replicated and two new original dimensionality reduction techniques were introduced. Yet, a great amount of other dimensionality reduction techniques and clustering methods could be tested in the future so as to outperform the results obtained in the present study. Interesting candidates would be HDBSCAN [67] for which, in some references, its transductive nature could be overcome by cross-validation (https://hdbscan.readthedocs.io/en/latest/prediction_tutorial.html, accessed on 23 November 2022). This method has the advantage of not tending to form Voronoi-cell-shaped clusters that tend to have similar numbers of samples per cluster (like DBSCAN). Such methods appear to be more adapted in the case of subgrouping samples in groups that can have diverging prevalence in the population.

Additionally, a very good semi-supervised candidate could be the heterogeneity through discriminative analysis (HYDRA) method [68], which has proven to be quite efficient at subtyping schizophrenia [69,70]. However, this method would be best fit for application to neuroimaging datasets, rather than questionnaire datasets.

## 5. Conclusions

The present study aimed at presenting a new semi-supervised framework to bring guidance when facing the issue of heterogeneity in the population of tinnitus patients. It is the first to achieve such a clustering while enabling quantified comparisons between the performance of different algorithmic combinations. Through this process and through a bootstrap procedure for stability assessment, a 20-cluster solution was selected and presented. With this solution, most clusters were confirmed by ENT tinnitus experts to convey strong clinical relevance. Such clusters define homogeneous subphenotypes of patients. Those are relevant to be studied independently to look for objective signatures of different potential underlying pathophysiological mechanisms.

## Figures and Tables

**Table 1:** Subgrouping rules’ semantic distinctions; approved excerpt from Genitsaridi, 2021.

Term	Definition
Subgroup	A part of a population (generic term)
Subphenotype	A part of a population with a distinct set of observable characteristics (based on Lötvall et al., 2011)
Subtype	A part of a population with a condition that can be used to guide treatment decisions (based on Saria and Goldenberg, 2015)
Endotype	A part of a population with a condition with distinct underlying mechanisms (based on Anderson, 2008; Lötvall et al., 2011)

**Table 2:** Sample characteristics’: quantitative features of the preprocessed database.

Sample Characteristics for Quantitative Features (N = 2772)					
	Mean	Std	Min	Max	Missing values (%)
Age (in years)	51.3	14.74	18	95	1.41
Tinnitus Handicap Inventory (THI)	50.98	23.83	0	100	4.4
Tinnitus duration (in months)	67.79	93.12	0	852	9.92
What percentage of time is tinnitus present (in %)?	66.34	28.19	0	100	16.31
VAS scale Tinnitus annoyance (0 to 10)	6.89	2.17	0	10	4.98
VAS scale Anxiety (0 to 10)	4.89	3.25	0	10	5.81
VAS scale Sleep quality (0 to 10)	5.25	3.35	0	10	6.28
VAS scale Hyperacusis (0 to 10)	5.05	3.41	0	10	5.77
VAS scale Vertigo (0 to 10)	2.36	2.94	0	10	5.77
VAS scale Headache (0 to 10)	2.16	3.23	0	10	0,29
VAS scale Jaw pain (0 to 10)	1.9	2.79	0	10	6.78
VAS scale Cervical pain (0 to 10)	2.63	3.39	0	10	0.4
Khalfa questionnaire score (hyperacusis)	19.72	9.73	0	42	11
HAD questionnaire on anxiety	9.18	4.24	0	21	12.59
HAD questionnaire on depression	6.47	4.6	0	21	12.73
Insomnia Sleep Index (ISI) score	12.39	6.62	0	28	8.44
Epworth scale score	7.62	4.7	0	24	7.65
Sleep onset latency (in min)	35.72	27.23	0	121	11.62
Abbreviations: HAD: Hospital Anxiety and Depression scale, VAS: Visual Analog Scale					

**Table 3:** Sample characteristics: categorical features of the preprocessed database.

Sample Characteristics for Categorical Features (N = 2772)				
Occurrences count	Yes/Choice 1	Sometimes/Choice 2	No/Choice 3	Missing values (%)
Gender (Choice 1: Female; Choice 2: Male)	1491	1226		1.95
Tinnitus apparition (Choice 1: brutal, Choice 2: progressive)	1665	1099		0.3
Tinnitus side (Choice 1: left, Choice 2: bilateral, Choice 3: right)	1015	910	760	3.14
Tinnitus lateralisation (1: bilateral, 2: partially lateral, 3: lateral)	910	743	1032	3.14
Tinnitus intensity (1: Low, 2: Medium; 3: Elevated)	1282	990	500	0.3
Was tinnitus caused by an acoustic trauma? (Yes, No)	460		2304	0.3
Is the tinnitus pulsatile? (Yes, No)	231		2533	0.3
Is the tinnitus a narrow band noise? (Yes, No)	283		2481	0.3
Do you have somato-sensory modulations? (Yes, No)	446		2318	0.3
Do you have jaw somato-sensory modulations? (Yes, No)	317		2447	0.3
Do you have neck somato-sensory modulations? (Yes, No)	256		2508	0.3
Do you have often headaches (Yes, No)	826		1938	0.3
Do you have often cervical pain/rigidity? (Yes, No)	1198		1566	0.3
Do you have trouble falling asleep? (Yes, Sometimes, No)	972	505	1134	5.81
Do you have nocturnal awakenings? (Yes, Sometimes, No)	425	530	1644	6.24
Are you feeling tired when awake? (Yes, Sometimes, No)	653	574	1360	6.67
Do you often snore? (Yes, No)	1152		1438	6.57
Do you have sleep apnea syndrome? (Yes, Probably, No)	217	238	2102	7.76
Normal hearing? (Yes, No)	337		2427	0.3
Sensorineural hearing loss (Yes, No)	1563		656	19.95
Transmissionnal hearing loss (Yes, No)	197		2022	19.95
Notch Hearing Loss (Yes, No)	251		1968	19.95
High Frequencies hearing loss (Yes, No)	146		2073	19.95
Cophosis (Yes, No)	38		2181	19.95

**Table 4:** Endotypes’ prevalence in the database.

Endotypes Prevalence in the Dataset (N=2772)		
Endotype	Occurrences	Percentage of the sample
Pulsatile tinnitus	231	8.33%
Otosclerosis	38	1.37%
Menière’s disease	35	1.26%
Neurinoma	24	0.87%
Petrous bone fracture	14	0.51%
Eustachian tube dysfunction	7	0.25%
**Total**	**349**	**12.59**%

**Table 5:** Range of hyperparameters.

Hyperparameters and Associated Grid Search Ranges			
Hyperparameter	Associated algorithm (s)	Range of exploration	Steps of exploration
Number of components	PCA, FAMD	[ 2, 20 ]	1
Number of components	t-SNE	[2, 6]	1
Perplexity	t-SNE	[5, 200]	5 by 5 to 40 then to 25 by 25 from 50 to 200
Number of clusters	K-means, DBSCAN *	[ 2, 20 ]	1
Epsilon	DBSCAN	[ 0, 10 ]	0,1
Minsample	DBSCAN	[ 2, 15 ]	1

* For DBSCAN, the number of cluster cannot be directly assigned, hence only the solutions in the range from 2 to 20 clusters were selected.

**Table 6:** Performance benchmark.

Algorithms Best Performances Comparison				
Dimensionality reduction method	Clustering algorithm	V-Measure (Beta = 0.1)	Stability assessment (AMI *)	Silhouette score
Principal Component Analysis	K-means	0.142	0.716	0.321
Principal Component Analysis	DBSCAN	0.035	0 clusters difference	−0.17
Factor Analysis of Mixed Data	K-means	0.146	0.707	0.324
Factor Analysis of Mixed Data	DBSCAN	0.049	0 clusters difference	−0.15
t-distributed stochastic neighbor embedding	K-means	0.381	0.728	0.351
t-distributed stochastic neighbor embedding	DBSCAN	0.346	0 clusters difference	0.008

* AMI: adjusted mutual information, calculated between k-means clustering, not possible for DBSCAN clusterings comparison.

**Table 7:** Characteristics of the clusters of the selected clustering. The pooled Cohen’s D was used as the effect size measurement. If a feature is present in the positive differentiation column, it means that the mean score of this feature for the patients in this cluster is more elevated than the mean of this feature for the rest of the patients, with the Cohen’s D effect size given in the associated effect size column. Conversely, if a feature is present in the negative differentiation column, it means that the mean score of this feature for the patients in this cluster is less elevated than the mean of this feature for the rest of the patients. As an illustration, in Cluster 2, THI is present in the positive differentiation feature. This means that the patients in Cluster 2 have a more elevated THI score than average, whereas if the feature age is present in the negative differentiation column, it means that the patients of Cluster 2 are younger than the rest of the tinnitus patients’ sample.

Individual Clusters Characteristics of the Finally Selcted Clustering						
Cluster Number	Cluster General Description	Number of Patients	Positive Differentiation		Negative Differentiation	
			Feature	Effect Size	Feature	Effect Size
1	Acoustic trauma without annoyance	83	Initial acoustic trauma	0.96	ISI	−1.3
			Progressive apparition	0.8	VAS Sleep quality	−1.2
			Tinnitus duration (in months)	0.71	Trouble falling asleep	−1.1
			Men	0.69	THI	−1.1
					Tired when awake	−1.0
					HAD anxiety	−0.98
					VAS Anxiety	−0.96
					Nocturnal awakenings	−0.86
					HAD depression	−0.84
					Brutal onset	−0.71
					VAS Tinnitus annoyance	−0.7
2	Notch hearing losses	91	Notch Hearing Loss	4.6	Sensorineural hearing loss	−1.5
			VAS Anxiety	0.56	Age (in years)	−0.73
			Trouble falling asleep	0.53	Tinnitus pulsatile	−0.32
			THI	0.49	Neck somato-sensory modulations	−0.31
			Tired when awake	0.43		
			HAD anxiety	0.41		
			ISI	0.35		
			VAS Sleep quality	0.33		
			Sleep onset latency	0.33		
3	Cophosis group	18	Cophosis	>10	Sensorineural hearing loss	−1.6
			Hyperacusis Khalfa	0.65	Initial acoustic trauma	−0.45
			VAS Tinnitus annoyance	0.64	Normal hearing	−0.38
			VAS Vertigo	0.63	Jaw somato-sensory modulations	−0.36
			Tinnitus lateralised	0.62	VAS Headache	−0.33
			THI	0.58	Notch Hearing Loss	−0.32
			Age (in years)	0.39	Tinnitus pulsatile	−0.31
			VAS Hyperacusis	0.39		
			Tinnitus duration (in months)	0.34		
			Nocturnal awakenings	0.33		
			% time tinnitus present	0.32		
4	Loud narrow band tinnitus in OSA elderly	57	Narrow band noise	2.7	Headaches	−0.34
			OSA	0.62	Notch Hearing Loss	−0.33
			High tinnitus intensity	0.54	VAS Headache	−0.32
			Age (in years)	0.52		
			VAS Tinnitus annoyance	0.47		
			Sensorineural hearing loss	0.45		
			Snoring	0.44		
			VAS Cervical pain	0.42		
			Cervical pain/rigidity	0.36		
			% time tinnitus present	0.33		
5	Right pulsatile tinnitus	28	Tinnitus pulsatile	3.8	Sensorineural hearing loss	−1.2
			Normal hearing	1.4	Snoring	−0.62
			Tinnitus side right	0.8	Age (in years)	−0.62
			Tinnitus lateralised	0.56	VAS Cervical pain	−0.62
			Notch Hearing Loss	0.31	Men	−0.54
					Cervical pain/rigidity	−0.53
					HAD depression	−0.49
					Initial acoustic trauma	−0.46
					Tinnitus duration (in months)	−0.39
					VAS Anxiety	−0.37
					THI	−0.34
					VAS Tinnitus annoyance	−0.33
6	Acoustic trauma with hearing loss	81	Initial acoustic trauma	1.4	Brutal onset	−0.59
			Sensorineural hearing loss	0.65	VAS Headache	−0.58
			Men	0.49	VAS Anxiety	−0.55
			Age (in years)	0.43	Headaches	−0.52
			Tinnitus duration (in months)	0.41	HAD anxiety	−0.5
			Progressive apparition	0.35	Tinnitus lateralised	−0.42
					VAS Sleep quality	−0.42
					Sleep onset latency	−0.42
					Somato-sensory modulations	−0.42
					Normal hearing	−0.4
					VAS Jaw pain	−0.4
7	Tinnitus and insomnia	90	Sensorineural hearing loss	0.63	VAS Headache	−0.58
			ISI	0.51	Headaches	−0.54
			VAS Sleep quality	0.51	Somato-sensory modulations	−0.46
			Tired when awake	0.42	Normal hearing	−0.4
			Nocturnal awakenings	0.38	Jaw somato-sensory modulations	−0.38
			Trouble falling asleep	0.37	Snoring	−0.38
					Neck somato-sensory modulations	−0.35
					Notch Hearing Loss	−0.34
					Narrow band noise	−0.32
					Tinnitus pulsatile	−0.32
					High Frequencies hearing loss	−0.31
8	Neck Somatosensory tinnitus	95	Neck somato-sensory modulations	5.3		
			Somato-sensory modulations	3.2		
			Jaw somato-sensory modulations	1.8		
			VAS Cervical pain	0.93		
			Cervical pain/rigidity	0.74		
			HAD anxiety	0.7		
			VAS Jaw pain	0.64		
			THI	0.6		
			VAS Vertigo	0.57		
			ISI	0.56		
			VAS Anxiety	0.55		
9	Pulsatile tinnitus	59	Tinnitus pulsatile	4.8	High tinnitus intensity	−0.53
			Trouble falling asleep	0.68	Men	−0.52
			Tinnitus lateralised	0.6	Initial acoustic trauma	−0.47
			Sensorineural hearing loss	0.54	Somato-sensory modulations	−0.35
			Sleep onset latency	0.39	Cervical pain/rigidity	−0.34
			Age (in years)	0.31	Normal hearing	−0.34
					Tinnitus duration (in months)	−0.32
					Jaw somato-sensory modulations	−0.31
10	High frequency hearing loss with tinnitus	85	High Frequencies hearing loss	8.5	Sensorineural hearing loss	−1.8
			Normal hearing	2.3	% time tinnitus present	−0.43
			Tired when awake	0.33	Age (in years)	−0.39
					Snoring	−0.32
					OSA	−0.31
11	Sudden hearing loss	82	Brutal onset	0.94	Progressive apparition	−0.8
			Sensorineural hearing loss	0.68	ISI	−0.53
			Tinnitus lateralised	0.55	Somato-sensory modulations	−0.46
			Headaches	0.39	VAS Jaw pain	−0.45
					Normal hearing	−0.4
					Sleep onset latency	−0.37
					Jaw somato-sensory modulations	−0.37
					HAD anxiety	−0.36
					Tired when awake	−0.36
					Neck somato-sensory modulations	−0.35
					Notch Hearing Loss	−0.34
12	Elderly people with bothersome tinnitus and OSA	61	OSA	1.8	Somato-sensory modulations	−0.41
			HAD depression	0.73	Normal hearing	−0.39
			Snoring	0.73	Jaw somato-sensory modulations	−0.37
			THI	0.73	Brutal onset	−0.34
			% time tinnitus present	0.69	Notch Hearing Loss	−0.33
			Hyperacusis Khalfa	0.63	Tinnitus pulsatile	−0.32
			VAS Tinnitus annoyance	0.62		
			Sensorineural hearing loss	0.62		
			Epworth	0.6		
			Age (in years)	0.59		
			VAS Anxiety	0.59		
13	Otosclerosis oriented group	43	Transmissionnal hearing loss	4.7	OSA	−0.43
			Progressive apparition	0.59	Brutal onset	−0.42
			Tinnitus pulsatile	0.56	Initial acoustic trauma	−0.4
			Tinnitus side right	0.47	Somato-sensory modulations	−0.38
			Tired when awake	0.39	Jaw somato-sensory modulations	−0.36
			VAS Sleep quality	0.35	Notch Hearing Loss	−0.33
			ISI	0.34	Normal hearing	−0.32
			VAS Vertigo	0.32		
14	Somatosensory tinnitus without annoyance	73	Somato-sensory modulations	2.8	THI	−0.79
			Jaw somato-sensory modulations	2.7	ISI	−0.75
			Neck somato-sensory modulations	1.1	VAS Sleep quality	−0.67
			Men	0.58	VAS Anxiety	−0.64
					VAS Tinnitus annoyance	−0.62
					HAD anxiety	−0.61
					Trouble falling asleep	−0.6
					Hyperacusis Khalfa	−0.55
					HAD depression	−0.54
					% time tinnitus present	−0.51
					VAS Hyperacusis	−0.46
15	Tinnitus and headaches	70	Headaches	1.2	Cervical pain/rigidity	−0.54
			VAS Headache	0.9	VAS Cervical pain	−0.53
			ISI	0.63	Somato-sensory modulations	−0.46
			THI	0.62	Normal hearing	−0.4
			Sensorineural hearing loss	0.6	Jaw somato-sensory modulations	−0.37
			VAS Sleep quality	0.6	Neck somato-sensory modulations	−0.35
			VAS Tinnitus annoyance	0.55	Notch Hearing Loss	−0.33
			% time tinnitus present	0.54	Tinnitus pulsatile	−0.32
			VAS Hyperacusis	0.43	High Frequencies hearing loss	−0.3
			Trouble falling asleep	0.42		
			Hyperacusis Khalfa	0.42		
16	Habituated patients	103	Sensorineural hearing loss	0.44	VAS Sleep quality	−1.2
					ISI	−1.1
					Tired when awake	−1.0
					THI	−0.92
					VAS Anxiety	−0.88
					Nocturnal awakenings	−0.87
					VAS Tinnitus annoyance	−0.86
					Hyperacusis Khalfa	−0.83
					HAD anxiety	−0.78
					Trouble falling asleep	−0.74
					VAS Hyperacusis	−0.72
17	Young people with normal hearing with bothersome tinnitus and hyperacusis	60	Normal hearing	2.0	Age (in years)	−1.3
			VAS Headache	0.46	Sensorineural hearing loss	−1.2
			HAD anxiety	0.44	Snoring	−0.48
			THI	0.43	Tinnitus lateralised	−0.42
			VAS Jaw pain	0.42	Initial acoustic trauma	−0.37
			VAS Hyperacusis	0.36	Notch Hearing Loss	−0.33
			Hyperacusis Khalfa	0.33	Tinnitus pulsatile	−0.32
			VAS Anxiety	0.33		
18	Tinnitus patients with depression	71	HAD depression	1.6	Somato-sensory modulations	−0.46
			THI	1.3	Normal hearing	−0.4
			Sleep onset latency	1.2	Jaw somato-sensory modulations	−0.37
			HAD anxiety	1.2	Neck somato-sensory modulations	−0.35
			VAS Anxiety	1.1	Epworth	−0.33
			ISI	1.1	Notch Hearing Loss	−0.33
			VAS Sleep quality	1.1	Tinnitus pulsatile	−0.32
			VAS Tinnitus annoyance	0.95	Narrow band noise	−0.3
			VAS Cervical pain	0.94	High Frequencies hearing loss	−0.3
			% time tinnitus present	0.83		
			Trouble falling asleep	0.81		
19	Head trauma tinnitus patients(Majority of petrous bone fractures)	58	VAS Headache	2.0	Progressive apparition	−0.52
			VAS Jaw pain	1.4	Men	−0.47
			Headaches	1.3	Somato-sensory modulations	−0.35
			VAS Vertigo	1.1	OSA	−0.34
			HAD anxiety	1.1	Neck somato-sensory modulations	−0.34
			HAD depression	1.1		
			THI	1.0		
			ISI	1.0		
			VAS Sleep quality	0.98		
			VAS Anxiety	0.96		
			VAS Cervical pain	0.92		
20	Tinnitus with OSA seemingly affecting middle ear conduction, not bothersome	77	Transmissionnal hearing loss	1.0	THI	−1.1
			Narrow band noise	0.86	VAS Sleep quality	−1.1
			OSA	0.56	ISI	−1.1
			Snoring	0.39	VAS Anxiety	−1.0
					Hyperacusis Khalfa	−1.0
					Tired when awake	−1.0
					VAS Hyperacusis	−0.99
					VAS Tinnitus annoyance	−0.84
					Trouble falling asleep	−0.81
					HAD depression	−0.76
					HAD anxiety	−0.71

THI: Tinnitus Handicap Inventory, ISI: Insomnia Severity Index, HAD: Hospital Anxiety and Depression scale score, OSA: Obstructive sleep apnea, VAS: Visual Analog Scale, Hyperacusis Khalfa: Khalfa questionnaire score on hyperacusis.

## Data Availability

The anonymized dataset is available upon request.

## Abbreviations

The following abbreviations are used in this manuscript:

THI	Tinnitus Handicap Inventory
VAS	Visual analog scale
HAD	Hospital Anxiety and Depression scale
ISI	Insomnia Sleep Index
ENT	Ear, nose, and throat
PCA	Principal component analysis
FAMD	Factor analysis of mixed data
t-SNE	t-distributed stochastic neighbor embedding
DBSCAN	Density-based spatial clustering of applications with noise
AMI	Adjusted mutual information
OSA	Obstructive sleep apnea
